# Conceptualising conceptual resilience. A comparative approach

**DOI:** 10.1007/s13347-026-01045-0

**Published:** 2026-02-28

**Authors:** Samuela Marchiori, Joseph Sta. Maria

**Affiliations:** Ethics and Philosophy of Technology, Delft University of Technology, Delft, The Netherlands

**Keywords:** Conceptual engineering, Conceptual disruption, Resilience, Conceptual resilience, Philosophy of technology, Comparative conceptual engineering

## Abstract

Much of the existing literature on conceptual engineering in the philosophy of technology has concentrated on identifying when and how concepts are disrupted under pressure, and how such disruptions can be addressed through conceptual engineering interventions. By and large, this literature has predominantly resorted to conceptual engineering as an approach to diagnose and remedy disruption. Recent work by Lundgren (2024) suggests that a shift from restorative to preventative conceptual engineering is warranted: rather than analysing disruptions post hoc, concepts can be deliberately designed to resist disruption from the outset. This paper introduces and develops the notion of *conceptual resilience* as the capacity of concepts to maintain continuous functional adequacy despite tensions, pressures, or other disturbances. Unlike Lundgren’s (2024) account, which frames this phenomenon in terms of *conceptual stability*, we argue that *resilience* better accommodates a broader range of modes of resistance to disruption, including those that involve adaptive transformation rather than static continuity. We further argue that conceptual resilience is not a binary property, but a capacity exhibited in degrees. Drawing from interdisciplinary literatures, we introduce two heuristic framings—*Conceptual Resilience as Immutability* (CRI) and *Conceptual Resilience as Adaptability* (CRA)—which capture contrasting yet complementary ways in which concepts preserve their functional adequacy under pressure.

## Introduction

Recent work on conceptual engineering in the philosophy of technology has increasingly turned its attention to the phenomenon of *conceptual disruption*, understood as the interruption in the functional adequacy of concepts^1^ (originally, Löhr, 2023; subsequently, Hopster & Löhr, 2023; Hopster et al., 2023; Marchiori & Scharp, 2024; van de Poel et al., 2023).^2^ On such an account, concepts have functions and are adequate to the extent that they fulfil their functions; conversely, concepts are disrupted when they fail to do so.^3^ For example, concepts can become contested (e.g., failing to serve as a shared epistemic tool, such as in the case of democracy; Gallie, 1955), misleading (e.g., introducing distortions into discourse, as with rigid conceptualisations of marriage that fail to accommodate social change; Anderson, 2013; Fineman, 2001, 2006), or otherwise defective (e.g., by being historically tainted, fuelling unrealistic expectations, distorting value judgments, and enabling conceptual appropriation, such as in the case of ARTIFICIAL INTELLIGENCE illustrated in Hopster, 2024a). This can lead, among others, to breakdowns in communication, epistemic confusion, and normative uncertainty (Hopster, 2024b).

Conceptual disruption is a central concern for the philosophy of technology and bears significance for our engagement with technology in at least two ways. First, technology has an impact on the socio-technical landscape in which it is designed, developed, and deployed. As recent discussions illustrate, such an impact extends to concepts themselves (e.g., Hopster & Löhr, 2023; Hopster et al., 2023; Löhr, 2023; Marchiori & Scharp, 2024; Nickel, 2020; van de Poel et al., 2023). Relatedly, when new technologies are introduced, they may prompt instances of conceptual disruption. For example, the advent of autonomous vehicles has led to (ongoing) sustained debates about the (in)adequacy of existing notions of responsibility (Goetze, 2022; Hindriks & Veluwenkamp, 2023; Matthias, 2004; Santoni de Sio & Mecacci, 2021). More recently, the proliferation of large language models has prompted renewed scrutiny of the adequacy of concepts related to writing (Floridi, 2025), authorship (Bajohr, 2024; Bimo, 2024; Coeckelbergh & Gunkel, 2025; Huang et al., 2025), and intellectual property (Gervais, 2025). Second, the very concepts that underpin and structure our analysis, evaluation, and governance of technology may be disrupted in a way that hinders our ability to do so effectively. Consequently, the relevance of conceptual disruption for the philosophy of technology extends beyond technology-induced disruptions alone.

The focus on conceptual disruption is proving useful as a diagnostic tool to signal that conceptual engineering (the evaluation, design, and implementation of concepts; Chalmers, 2020; Isaac et al., 2022) may be warranted. Much of the existing (albeit still limited) literature on conceptual disruption has pursued this diagnostic function in tandem with a curative orientation, proposing strategies for restoring conceptual adequacy through conceptual engineering interventions once disruption has occurred (Hopster & Löhr, 2023; Hopster et al., 2023; Löhr, 2023; Marchiori & Scharp, 2024; van de Poel et al., 2023). These approaches have yielded valuable insights into how and why concepts are disrupted, and into how such a disruption may be overcome, whenever deemed desirable. Yet, to the extent that the literature on conceptual disruption has so far predominantly linked the investigation of when and how concepts are disrupted under pressure to restorative approaches, it has largely overlooked the complementary dimension of how concepts can resist disruption. Relatedly, the investigation of how concepts remain (more or less) functionally adequate *under* pressure remains widely under-explored. This asymmetry invites a complimentary line of inquiry.

This paper takes up this invitation. That is, there may be a benefit in articulating how concepts can resist pressures and disturbances (technology-induced or otherwise), beyond how they may fail to do so. Moreover, if there is a strong sense in which conceptual disruption may be undesirable, as has been suggested by Lundgren (2024),^4^ further investigating how concepts may be more or less equipped to deal with disturbances and resist conceptual disruption should be of interest to conceptual engineers. To this end, the paper introduces the notion of *conceptual resilience*, understood as the ability of concepts to resist conceptual disruption by maintaining continuous functional adequacy despite tensions, pressures, or other disturbances. This builds on and expands recent work by Lundgren (2024) on the notion of *conceptual stability*.^5^ While Lundgren (2024) advocates for static resistance to disruption through stability, this paper also accounts for a dynamic mode of resilience through adaptability. In doing so, the paper provides a more comprehensive illustration of how concepts remain adequate under pressure, thus refining the tools available to conceptual engineers for engaging with conceptual adequacy under conditions of disturbances. Importantly, the account of conceptual resilience developed in this paper is non-committal on the desirability of (re-)engineering for resilience, and rather provides a basis from which both advocates and critics of resilience can evaluate its desirability.

The paper is structured as follows. Section 2 illustrates the notion of conceptual stability as introduced by Lundgren (2024) and argues for a move from stability to the more comprehensive notion of conceptual resilience, of which stability is a facet. Section 3 proposes that conceptual resilience should not be understood as a binary property, but as a capacity that can be exhibited in degrees. Section 4 articulates how concepts can exhibit resilience by distinguishing between different modes of resilience, drawing from interdisciplinary work to introduce two heuristic framings—Conceptual Resilience as Immutability (CRI) and Conceptual Resilience as Adaptability (CRA)—which illuminate contrasting yet complementary modes through which concepts preserve functional adequacy under pressure. Section 5 contains the conclusion.

## From Conceptual Stability to Conceptual Resilience

Much of the existing literature on conceptual disruption has concentrated on identifying when and how concepts are disrupted under pressure, and how such disruptions can be addressed through conceptual engineering interventions (Hopster & Löhr, 2023, Hopster et al., 2023; Löhr, 2023; Marchiori & Scharp, 2024). Recent work by Lundgren (2024) shifts the attention from restorative to preventative conceptual engineering. Rather than analysing conceptual disruptions after they occurred, Lundgren suggests that some concepts can be deliberately designed to resist disruption in the first place. Lundgren observes that technological developments, normative critique, and conceptual challenges frequently force revisions or replacements of concepts. If conceptual engineers could construct concepts that resist such pressures, the need for future revisions might be mitigated or even eliminated.

Lundgren (2024) frames the discussion in terms of conceptual *stability*, suggesting that some concepts can be designed in ways that ensure they remain stable across contexts, avoiding counterexamples, normative critique, and conceptual disruption.^6^ Specifically, Lundgren proposes that this can be achieved by formulating concepts at a sufficiently high level of abstraction. His case study of information security illustrates this strategy: rather than defining security in terms of fixed properties that may be challenged by technological developments, the “appropriate access” definition relativises security to stakeholder needs, ensuring that the concept remains applicable even as specific security concerns evolve. Following Lundgren’s account, conceptual *stability* refers to the preservation of a concept’s functional adequacy across different contexts through preemptive (re-)design.

In this paper, we build on and extend Lundgren’s (2024) proposal by offering a more comprehensive and explanatorily rich account of the ability of concepts to resist conceptual disruption. Indeed, while Lundgren’s model provides a compelling account of how some concepts can be (re-)engineered in such a way that makes them resistant to disruption, it does not fully capture the broader range of ways in which concepts remain adequate despite disturbances. Specifically, we argue that *conceptual stability* and *conceptual disruption* alone cannot fully capture the spectrum of ways in which concepts can be affected by tensions, pressures, and other disturbances, which need not raise to the level of a conceptual disruption to be of interest to conceptual engineers. A richer understanding should be explored of how concepts behave under pressure, providing the necessary vocabulary to describe not just how concepts are disrupted under pressure, but how well they can endure such pressures and disturbances while retaining functional adequacy.

With such concerns in mind, we hereby introduce the notion of *conceptual resilience* to describe the ability of concepts to resist conceptual disruption by maintaining continuous functional adequacy despite tensions, pressures, or other disturbances.^7^*Despite tensions*,* pressures*,* or other disturbances* is crucial here. Indeed, a concept may be functionally adequate and yet not well-suited to resist pressures. For example, consider a concept that is adequate because it has never been challenged, but would not be able to maintain functional adequacy if challenged.^8^ There seems to be a salient difference between having the capacity to resist conceptual disruption and not having been disrupted.^9^

Consider the concept of prime number. A number *p* is said to be prime if (i) *p* > 1, and (ii) *p* has no positive divisors except 1 and *p* (Hardy & Wright, 1960, p. 2). That is, primes are defined as numbers that can only be divided by themselves and by 1. While a great deal of research has been (as is still being) conducted on primes, such as research aimed at understanding what primes are (e.g., Jameson, 2003; Maynard, 2019; Saidak, 2006) and how they are distributed (e.g., Conrey, 2003; Tao, 2011), developing new mathematical methods to study them (e.g., Selberg, 1949), applying them in cryptography (e.g., Ivy et al., 2012; Shemanske, 2017; Zhou & Tang, 2011), and exploring connections between primes and other advanced areas of mathematics (e.g., Lozano-Robledo, 2019; Nathanson, 1996), such a concept remains functionally adequate and (to the best of our knowledge) unchallenged in mathematics scholarship.^10^

Thus, resisting disruption is alone insufficient for the attribution of conceptual resilience, as resilience must account for the concept’s capacity to adequately fulfil its function(s) under strain. In this sense, conceptual resilience is best understood as a disposition rather than a static state, and more is needed to determine whether a concept is resilient. In particular, the presence of disturbances is essential for the assessment of whether concepts are resilient.

We propose that framing the phenomenon under investigation in terms of *resilience* rather than *stability* better accommodates the plurality of conceptual responses to pressures and disturbances, particularly those that involve adaptive transformation rather than static continuity (as in Lundgren, 2024), a phenomenon that the notion of stability may overlook. The concept of resilience has been extensively theorised across multiple disciplines, where it generally denotes the capacity of a system, entity, or structure to withstand, absorb, or recover from external pressures and disturbances. In ecology, resilience is often understood as the ability of an ecosystem to return to equilibrium after perturbation, either by resisting change (engineering resilience) or by adapting to new conditions (adaptive resilience) (Holling, 1973). Psychology and cognitive science, in turn, frame resilience as an individual’s ability to cope with adversity, maintaining psychological stability or adjusting effectively to stressors (Bonanno, 2004). In engineering, resilience is associated with fault tolerance and robustness, referring to the ability of structures or systems to continue functioning despite failures or unexpected disruptions. This broader theoretical background informs our account of *conceptual* resilience.

The distinction between *conceptual stability* and *conceptual resilience* is not merely terminological but reflects a substantive theoretical commitment. If we were to speak only in terms of conceptual stability, we would risk overlooking cases where concepts persist by adapting to disturbances rather than by resisting such disturbances. Our approach, therefore, does not reject conceptual stability but subsumes it as one possible mode of conceptual resilience. While some concepts achieve resilience through stability, others achieve it through flexibility, and our account seeks to make this plurality of modes of resilience explicit. Unlike *stability*, which suggests a continuation of the same state, *resilience* encompasses continuous functional adequacy as both persistence and adaptability. Framing the discussion in terms of conceptual resilience rather than stability allows us to recognise that not all concepts endure by remaining fixed—many persist precisely because they mutate and adapt in response to pressures without losing their function, and without the need for conceptual engineering interventions. Ultimately, the advantage of this framework is that it enables conceptual engineers to assess not only *whether* a concept is resilient but *how* it exhibits resilience, facilitating a more nuanced evaluation of conceptual adequacy over time, and providing insights into the considerations that should guide conceptual engineering projects aimed at the (re-)design of concepts capable of resisting disruption.

In the next section, we build on the notion of conceptual resilience and explore the continuum of conceptual resilience, illustrating how some concepts exhibit resilience in degrees, as opposed to in binary terms. This will set the stage for the heuristic distinction between the different modes of conceptual resilience (Conceptual Resilience as Immutability and Conceptual Resilience as Adaptability), which we develop in detail in Sect. 4.

## A Graded Account of Conceptual Resilience

In the previous section, we introduced the notion of conceptual resilience to describe the ability of concepts to remain functionally adequate despite tensions, pressures, or other disturbances. Here, we expand on one feature of conceptual resilience, namely its non-binary nature. We argue that resilience should not be understood as an all-or-nothing property, but rather as a matter of degrees.

Conceptual stability, as described by Lundgren (2024), focuses on cases of maximal resistance to disruption. According to such a position, either a concept resists disruption or it does not. Indeed, as illustrated in the previous section, Lundgren (2024) introduces the notion of *conceptual stability* to capture both “*undisruptable concepts* (i.e., concepts that cannot be disrupted), and *stable concepts* (i.e., concepts that remain stable in a situation of disruption)” (p. 1). For the purposes of his paper, Lundgren (2024) explicitly treats undisruptable and stable concepts as interchangeable. A charitable reading of this move suggests that conceptual stability is conceived as a binary property rather than a gradable one. While one might, in principle, distinguish between degrees of stability, e.g., between concepts that are contingently stable under certain pressures and those that are stably adequate across all conditions, the notion of *undisruptability* allows no such gradation. Therefore, to equate the two is to align stability with a property that admits only of all-or-nothing states. This suggests a certain absoluteness in concepts’ functional adequacy: stable concepts are *disruption-proof*.^11^

We suggest that a similar binary classification should not be generalised beyond conceptual stability to conceptual resilience. Rather, it seems preferable to understand conceptual resilience as the property of being disruption-*resistant*, not disruption-*proof*. What this means in practice is that stable concepts as described by Lundgren (2024) will display the highest possible degree of resilience according to our framework, but being disruption-proof will not be the measure of conceptual resilience. This allows for a nuanced understanding of resilience that goes beyond a binary approach of resilient versus non-resilient concepts. Concepts displaying a low degree of resilience are significantly vulnerable to disruptions and can easily become obsolete or inadequate in the face of changing circumstances, while concepts with a high degree of resilience can withstand significant disturbances and still continue to adequately fulfil their functions.

Consider the concept privacy. Privacy extends far beyond the confines of individual data protection; it also includes decisional privacy, spatial privacy, and contextual integrity, each of which may be differentially impacted by technological, legal, and cultural developments (Nissenbaum, 2009, 2011, 2018; Solove, 2010). Other examples include autonomy, whose applicability varies across medical, legal, and technological contexts (Dworkin, 1988; Mackenzie & Stoljar, 2000); justice, which takes on different dimensions depending on its function in (among others) distributive or corrective frameworks (Rawls, 1971; Young, 1990); and transparency, which ranges in depth and clarity depending on the institutional or epistemic context (Ananny & Crawford, 2018; Floridi, 2023). This also suggests that conceptual resilience may be evaluated along several dimensions. For example, one might imagine that conceptual resilience may be assessed, beyond the ability of a concept to preserve functional continuity, through a contextual dimension, assessing the range of situations across which the concept remains functionally adequate. Indeed, a concept might be resilient by only retaining functional adequacy within a narrower range of contexts. In this sense, consider the legal concept liability, which was supplemented by the new notion strict liability, introduced to ensure accountability in cases where intent or negligence could not or need not be proven, e.g., with regards to pet owners’ liability for damages caused by their pets (Comporti, 2009), and which is currently being discussed as a liability regime for artificial intelligence systems and other emerging technologies (Čerka et al., 2015; de Fonseca et al., 2023; McDonald, 2023; Wendehorst, 2020). This addition preserved the original function of liability (allocating responsibility) while narrowing its scope of application after introducing strict liability to apply to circumstances to which the concept liability originally applied. The concept of liability thus retained functional adequacy but within a smaller contextual range. Relatedly, assessing resilience may involves recognising trade-offs between maintaining functional adequacy and retaining breadth of application, which may vary depending on a combination of the concept, the context of use, and the users.^12^ This seems to support the benefits of a graded model of resilience, whereby concepts lie on a continuum extending from a concept being disrupted to a concept being disruption-proof.

Moreover, practical considerations suggest that a graded approach to conceptual resilience is desirable for conceptual engineers. Indeed, conceptual engineering is a normative approach to conceptual work aimed at improve concepts, but such improvement typically proceeds by degrees. That is to say, when intervention is warranted,^13^ conceptual engineers rarely (if ever) (re-)engineer concepts so that they are *perfectly* able to fulfil their functions; rather, improvements are often incremental.^14^ Relatedly, assuming (as we do) that a notion able to pick out a concept’s ability to resist disruption will be of interest to conceptual engineers (e.g., for evaluative and design purposes), it seems misguided to expect that conceptual engineers will find such a notion useful when it only picks out the exceptional cases where concepts are disruption-proof. Holding too high a bar for conceptual resilience by only allowing to pick out concepts where the property of resisting disruption is exhibited in the highest degree would overly restrict the benefits of having such a notion in the first place. Indeed, this would reduce the practical utility of referring to resilience enough as to make such a notion uninteresting for the purpose of most conceptual engineering projects. Just like conceptual adequacy is almost exclusively assessed in relative terms (e.g., Nado, 2021; Marchiori, 2025), it seems plausible to expect that a useful notion of conceptual resilience will pick out concepts exhibiting varying degrees of resistance to disruption.

In this sense, understanding conceptual resilience as a matter of degree, with some concepts being more or less resilient depending on how well they perform their functions under pressure, is particularly important in the context of conceptual engineering. Identifying the degree to which concepts exhibit resilience enables conceptual engineers to assess their relative vulnerability to disruption and, in turn, develop more effective strategies for maintaining their functionality. A nuanced, gradated understanding of resilience thus facilitates a more targeted approach to conceptual (re-)design and conceptual engineering as a whole, ensuring that challenges, while inevitable, do not necessarily become catastrophic.

## Modes of Resilience. How do Concepts Exhibit resilience?

In the previous sections, we have proposed to conceptualise *conceptual resilience* as the capacity of concepts to maintain their functional adequacy despite tensions, pressures, or other disturbances. We have also argued that resilience should be understood as a property that admits of degrees. Together, these considerations provide valuable guidance for conceptual engineering, in that they equip conceptual engineers with a starting point for the identification, assessment, and (re-)design of resilient concepts.

However, the question remains open of *how* concepts exhibit resilience. That is, is a concept resilient because it maintains its functional adequacy by being impervious to disturbances, or because it preserves its functional adequacy by being malleable and adaptable in response to disturbances? An explicit articulation of the modes in which resilience manifests vis-à-vis concepts could yield a richer understanding of the phenomenon of conceptual resilience. Such clarity could in turn inform conceptual engineering interventions, by providing valuable insights for conceptual engineers seeking to design or refine concepts for (or against) resilience. Once more, we explore whether similar investigations into different modes of resilience are present in neighbouring literatures discussing related notions of resilience and, if that is the case, whether further clarity can be gained by tapping into such insights.

Drawing from, and synthesising, the rich tapestry of conceptualisations of resilience,^15^ two understandings of resilience often emerge, which reflect two approaches to the same notion, namely resilience as immutability and resilience as adaptive capacity (Gunderson, 2000). Among the facets of resilience as immutability are: (a) persistence, i.e., the ability to persist in the face of disturbance without altering the core function; and (b) resistance, i.e., the capacity to withstand disturbances without significant change (Holling, 1973; Pimm, 1984; Gunderson, 2000; Alexander, 2013). Among the dimensions of resilience as adaptative capacity are: (a) adaptation, i.e., the capacity to adapt to new conditions and evolve in response to disturbances (Rutter, 1987; Berkes & Folke, 1998; Luthar et al., 2000; Carpenter et al., 2001; Rose, 2004; Walker et al., 2004; Vogus & Sutcliffe, 2007; Hill et al., 2008; Norris et al., 2008; Folke et al., 2010; Magis, 2010); (b) coping, i.e., the implementation of short-term strategies to manage the immediate effects of disturbances (Southwick & Charney, 2012); (c) flexibility, i.e., the ability to change processes or structures in response to disturbances (Briguglio et al., 2009; Keck & Sakdapolrak, 2013); (d) learning, i.e., the process of learning from disturbances to improve future resilience (Southwick & Charney, 2012); (e) recovery, i.e., returning to a prior state after disturbance (Gunderson, 2000; Carpenter et al., 2001; Rose, 2004, 2007; Hill et al., 2008; Magis, 2010; Manyena et al., 2011); (f) redundancy, i.e., having multiple pathways or components to ensure function is maintained if one part fails; and (g) transformation, i.e., the potential to transform in order to maintain or improve a function in the face of disturbances (Folke et al., 2010; Westley et al., 2011). Lastly, one facet, that of anticipation, i.e., the ability to foresee potential disturbances and prevent loss of function through mitigation and proactive measures, is shared by both approaches (Hamel & Välikangas, 2003).^16^

Here, we build on this intuition and suggest that a similar distinction between immutability and adaptive capacity can be productively applied to the phenomenon of *conceptual* resilience. We propose that resilience vis-à-vis concepts could be meaningfully conceptualised as a phenomenon that can be framed in terms of immutability and adaptability, relative to concepts’ functional adequacy. We argue that such ways of framing conceptual resilience can be helpful heuristics for understanding how concepts exhibit resilience and, relatedly, can provide helpful pointers with respect to how to assess and (re-)engineer for resilience. On the one hand, conceptual resilience may manifest in static terms. We call this *Conceptual Resilience as Immutability* (CRI). According to CRI, conceptual resilience refers to the ability of concepts to remain unchanged, i.e., to retain continuity in how they perform their functions in the face of disturbances. Lundgren’s (2024) framework aligns closely with CRI, emphasising how concepts can be (re-)engineered to avoid disruption. On the other hand, conceptual resilience may manifest in dynamic terms. We call this *Conceptual Resilience as Adaptability* (CRA). According to CRA, conceptual resilience refers to the capacity of concepts to adapt and respond to disturbances while maintaining their core functions.^17^ Both facets of resilience will be illustrated in turn.

### Conceptual Resilience as Immutability (CRI)

A static account of conceptual resilience, associated with the notion of Conceptual Resilience as Immutability (CRI), emphasises the ability of a concept to remain unchanged despite external pressures. From this perspective, a concept is resilient if it can preserve its function(s) across different contexts and over time. This approach aligns with philosophical traditions that value stability and continuity in conceptual frameworks. In some cases, the immutability of a concept is necessary for it to serve as a reliable standard or foundation within a given system. For example, in legal contexts, the concept contract may need to remain relatively stable to provide a consistent basis for contractual obligations and rights. Here, the resilience of the concept is demonstrated by its ability to resist redefinition or reinterpretation, which could undermine its role in guiding legal reasoning and practice. In this static model, resilience is linked to the concept’s capacity to maintain its functional role without requiring modification. The stability of the concept ensures that it continues to meet the needs of its users, providing a reliable framework for thought, communication, or action. However, the functional account also recognises that this immutability must be justified by the concept’s continued functional adequacy. If a concept becomes outdated or irrelevant due to its rigidity, it would no longer be considered resilient from a functional perspective, as it would fail to fulfil its intended role effectively.

To better understand Conceptual Resilience as Immutability (CRI), consider the concept good as understood in the Platonic tradition. The concept good is often thought to be resilient because it continues to fulfil its normative function: guiding action and evaluating desirability across different contexts. But what grounds this resilience? The Platonic tradition provides one answer by positing that the concept good refers to a timeless and unchanging ideal (the *Form of the Good*) which subsumes all instances of goodness under a unified essence. According to this view, the concept is equipped with an intention or definition that pre-emptively includes all possible valid occurrences of its extension. This capacity to encompass all relevant instantiations is precisely what secures its ongoing functional adequacy.

On such a view, the concept is or becomes immutable: if its definition is already complete, there is no need to revise it in the face of new circumstances. The concept thereby remains adequate not because it adapts, but because it never encounters a challenge that calls for adaptability. Resilience here is thus not dynamic but static: the concept retains its function over time by virtue of not requiring change. In this way, its immutability is a feature, not a limitation, one that ensures continued use without the risk of disruption.

This understanding is echoed in Lundgren’s (2024) account of conceptual stability. Lundgren proposes that a concept such as “appropriate access” can be deliberately engineered to remain stable by formulating it at a high level of abstraction. This strategy aims to insulate the concept from disruption by counterexamples or technological shifts. The concept retains its relevance precisely because it resists revision, thus demonstrating a form of resilience as immutability.

Both the Platonic and Lundgren’s (2024) accounts point to an understanding of conceptual resilience where unchangeability is a strength, not a defect. These approaches suggest that when a concept’s function is sufficiently general and its scope carefully designed, it may remain stable across contexts and over time. This is the hallmark of CRI: functional adequacy secured through constancy rather than adaptability.

### Conceptual Resilience as Adaptability (CRA)

A dynamic account of conceptual resilience, associated with Conceptual Resilience as Adaptability (CRA), emphasises the concept’s ability to evolve and adapt in response to changing circumstances. Under this model, a concept is resilient if it can incorporate new information, adjust its boundaries, or modify its application to maintain its relevance and effectiveness. From a functional perspective, a dynamic account of resilience is particularly suited to contexts where flexibility and adaptability are key to maintaining functional adequacy. Concepts operating within rapidly changing environments, such as technology ethics or environmental policy, may require frequent adjustments to remain effective. For example, the environmental ethics concept sustainability may need to evolve in response to new scientific insights or societal shifts. Here, resilience is demonstrated by the concept’s capacity to adapt while continuing to guide action and decision-making effectively.

Consider once more the concept good. Unlike the Platonic tradition, which posits a fixed, essentialist definition of good that captures its extension through immutable criteria, the Confucian tradition departs from Platonic essentialism and offers a dynamic, context-sensitive understanding of good. In Confucian thought, the concept good—as expressed through the notion of Ren (仁), which can be understood as goodness, humanness, or benevolence—does not rest on a fixed, exhaustive definition.^18^ Rather, it is characterised by its open-endedness, and its resilience stems from its capacity to adjust to varying normative demands.

Unlike the Platonic approach, Confucius did not attempt to articulate a singular, universal definition of Ren. When his students asked about its meaning, he offered distinct descriptions tailored to their moral development and contextual circumstances.^19^ He described goodness as “hesitancy in speaking” to one disciple, “caring for others” to another, and “ritual propriety” in yet another instance. He also adapted to his interlocutors (Ashmore, 2004; D’Ambrosio, 2016, 2019). For example, when talking to Sima Niu, Confucius describes goodness as hesitancy in speaking because he believed the former’s loquaciousness to be detrimental to moral development. This pedagogical strategy reflects more than rhetorical flexibility: it embodies a conception of good that adapts to particularities rather than relying on generality.^20^ The absence of a fixed definition does not weaken the concept’s function; instead, it allows it to remain normatively effective across situations by modulating its application without compromising its role.

This adaptability is central to what we term Conceptual Resilience as Adaptability (CRA). The Confucian model suggests that a concept can remain resilient not by resisting change, but by incorporating it. The concept good, understood through Ren, is structurally open-ended, which allows it to accommodate emerging circumstances while continuing to provide moral guidance. In this way, resilience is achieved not through rigidity but through flexibility, not through preemptive completeness but through ongoing responsiveness. Importantly, this adaptability is not arbitrary. Confucius’ descriptions of Ren are always contextually anchored and morally instructive, aimed at cultivating virtue in line with his students’ specific situations. The lack of an exhaustive definition enables the concept to remain functionally adequate, because its normative orientation is preserved even as its instantiations shift. Thus, the Confucian approach illustrates how a concept can remain resilient by adapting rather than remaining unchanged. It maintains its function through situated elasticity, exemplifying CRA. This adaptive form of resilience demonstrates how a concept’s openness to re-articulation can itself be a strategy for enduring relevance and normative effectiveness.

### Relation between CRI and CRA

It should be emphasised that, by shedding light onto resilience as immutability and resilience as adaptability, we do not wish to argue that such facets should pick out a dichotomy. Importantly, such ways of framing resilience are neither to be taken as mutually exclusive nor jointly exhaustive to describe the ways in which concepts may resist conceptual disruption.

First, such facets are not mutually incompatible, and it is possible that the same instance of conceptual resilience may be described as CRI or CRA by different actors. For example, as Jacobs (1999) suggests in his analysis of the concept of sustainable development, some concepts may remain resilient not through pure immutability or adaptability, but through a layered structure combining a robust normative core with flexible operational features. This dual structure enables such concepts to absorb pressures without losing coherence. Ultimately, what we wish to highlight when drawing the distinction between a static and a dynamic understanding of resilience is the extent to which one’s perspective may greatly impact and shape how one understands and approaches resilience in the first place.

**Fig. 1:**
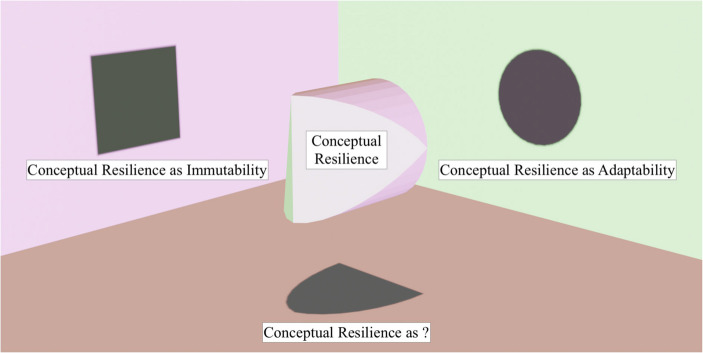
Facets of conceptual resilience

Second, CRI and CRA are not jointly exhaustive. While the literature converges significantly on the static and dynamic nature of resilience as a helpful heuristic through which to understand the notion of resilience, our focus on static and dynamic conceptual resilience does not exclude that additional facets of conceptual resilience may emerge in the future, which may be of interest to conceptual engineers. As such, our conceptualisation is not rigid with respect to the essential features of resilience. Were additional facets of resilience to come to the fore, which would change or refine our understanding of the concept and phenomenon itself, our framework already accounts for and accommodates such a contingency (see Fig. 1).

Ultimately, the static and dynamic framings of resilience may be mapped onto the continuum of resilience illustrated in Sects. 2 and 3, such that concepts exhibit resilience in degrees (low to high), and such resilience can be meaningfully understood through different frames (e.g., Conceptual Resilience as Immutability and Conceptual Resilience as Adaptability).

## Conclusion

Recent work on conceptual engineering in the philosophy of technology increasingly focuses on conceptual disruption, understood as an interruption in functional adequacy of concepts following tensions and disturbances affecting them, and the need for conceptual engineers to remedy such disruptions. This paper addressed a complementary and neglected phenomenon, which we referred to as conceptual resilience, i.e., the ability of concepts to maintain continuous functional adequacy despite tensions, pressures, or other disturbances. Contrary to existing work, which conceives resistance to disruption in absolute terms, we argued that conceptual resilience is best understood as a property that admits of degrees of resistance to disruption.

While such insights allow for intuitive assessments of whether a concept is (more or less) resistant to disruption, they do not, on their own, suffice to explain how a concept exhibits this property. To address this gap, we turned to neighbouring literatures that grapple with similar tensions, often framing resilience either as robustness, i.e., resistance to change, or as adaptability, i.e., flexible response to challenges. These distinctions, widely acknowledged in the literature on resilience, map well onto our heuristic of conceptual resilience, understood as resistance to disruption through immutability and adaptability.

Accordingly, we introduced two heuristic framings: Conceptual Resilience as Immutability (CRI) and Conceptual Resilience as Adaptability (CRA). Importantly, we do not consider such static and dynamic framings of resilience as mutually exclusive, nor as jointly exhaustive. Rather, we argue that resilient concepts can simultaneously exhibit both immutability and adaptability, depending on the framing of one’s analysis, and that additional facets of conceptual resilience may emerge in future research. The concept good provides a compelling example of how the same concept can be interpreted as embodying both static and dynamic resilience, depending on one’s framing. On the one hand, the concept’s resilience can be construed in terms of the immutability of its essential definition, which embodies a stable, unchanging essence that persists across different contexts and times. On the other hand, the concept’s resilience can be considered as a corollary of the concept’s ability to adapt, by virtue of its lack of exhaustive definition, to its various instantiations.^21^

Ultimately, what we aim to show is that conceptual engineers can fruitfully draw upon the conceptualisation of resilience that is prevalent in domains beyond the philosophical one. Consistently with the broader literature on resilience, we suggested that different approaches to understanding how concepts avoid disruption (through immutability or adaptability) should be seen not as distinct *types* of resilience, but as complementary analytic *lenses* or *frames* through which one can illuminate different dimensions of a unitary phenomenon.

In introducing the notion of conceptual resilience for conceptual engineering, the paper further clarified what it means to prevent, not merely repair, conceptual disruptions. It also provided practical insights into how concepts can be (re-)designed to avoid future disruptions. While doing so, the paper has remained agnostic with regards to the desirability of (re-)engineering for concepts resilience. Future work will build on these insights to address the issue of desirability: should conceptual engineers aim to (re-)engineer concepts *for* resilience and, if so, under what conditions?

## Data Availability

Does not apply.

## Footnotes

1 The account of conceptual resilience advanced in this paper is intentionally non-committal regarding which view of concepts one ought to adopt. Rather than adopting a substantive metaphysical account of what concepts are, this paper recognises that competing theories of concepts (e.g., definitional, inferential, prototypical) are often motivated by distinct explanatory aims and background assumptions (Margolis & Laurence, 2023). Committing to any one of these would entangle the paper in debates that are tangential to its purpose. Instead, the discussion proceeds from a minimal and broadly compatible assumption: that concepts can be treated as functional kinds (Lalumera, 2009). On this view, concepts are individuated and re-identified by their functions. This functional account is methodologically justified in at least two ways. On the one hand, the appeal of this view lies in its compatibility with a range of competing theories of concepts. This allows the discussion to proceed while remaining neutral across rival theories of concepts and still providing a clear criterion for conceptual identity and conceptual change. On the other hand, understanding concepts as a functional kind also aligns the present discussion on conceptual resilience with existing accounts of conceptual disruption, which are themselves framed in functional terms (Hopster & Löhr, 2023; Hopster et al., 2023; Löhr, 2022, 2023; Marchiori & Scharp, 2024). Thus, adopting a functional view of concepts provides continuity between the discussions on disruption and resilience: if disruption marks the loss of functional adequacy *due to* pressures, resilience marks its preservation *despite* pressures. In this sense, the functional individuation of concepts is particularly well suited for articulating the dynamics of both conceptual disruption and conceptual resilience.

2 Different accounts of conceptual disruption have been put forth in the literature (Hopster & Löhr, 2023; Hopster et al., 2023; Löhr, 2022, 2023; Marchiori & Scharp, 2024), all of which are compatible with, if not explicitly endorsing, a functional approach. Here, we choose to draw from Marchiori & Scharp’s (2024) account of conceptual disruption as an “interruption in the […] functioning of concepts” (Marchiori & Scharp, 2024, p. 1). On this account, conceptual disruption can take two main forms (conceptual gaps and conceptual conflicts) and can vary in severity (from mild to severe) depending on how urgently it calls for a conceptual engineering intervention. *Conceptual gaps* arise when no existing concept adequately applies to a phenomenon that ought to be captured at a certain level of precision. Conversely, *conceptual conflicts* occur in three main ways, as inconsistencies (i) among concepts’ constitutive principles, (ii) between concepts’ constitutive principles and facts about the world, or (iii) between concepts’ constitutive principles and values or social norms (a *conceptual misalignment*, following Hopster & Löhr, 2023). Moreover, gaps and conflicts are not static, but may occur at different times in the very same process of conceptual disruption and develop into one another. Specifically, conceptual conflicts may be followed by conceptual gaps. For example, when new science and technology is introduced, this may lead to conceptual conflicts when existing concepts clash with new facts or practices. This may prompt attempts to redefine or adjust such concepts, which in turn may generate a gap, requiring the introduction of a new concept altogether. Conversely, a gap may reveal deeper tensions within our conceptual framework that eventually manifest as a conflict. For example, the invention of the mechanical ventilator first led to a conflict between the concepts life and death, which was later followed by a conceptual gap that led to the creation of the new concept brain death. Following Marchiori and Scharp (2024), conceptual disruption affects concepts both individually and collectively and may manifests differently depending on the level at which it occurs: at the level of individual concepts, clusters of related concepts, or the broader conceptual scheme as a whole. For the purpose of this paper, we will focus our attention on conceptual disruption affecting individual concepts.

3 It should be noted that which functions concepts (should) fulfil, as well as how to assess adequate functional fulfilment, are the object of ongoing discussion in the conceptual engineering literature (e.g., Cappelen, 2018; Cappelen & Plunkett, 2020; Köhler & Veluwenkamp, 2024; Queloz, 2019, 2022; Riggs, 2021; Thomasson, 2021, 2022). The literature has offered divergent accounts of conceptual functions, including but not limited to: proper functions (Millikan, 1989), etiological functions (Prinzing, 2018; Simion & Kelp, 2020), system functions (Haslanger, 2020; Thomasson, 2020), practical, evaluative, animating, and inferential functions (Queloz, 2019), and normative functions (Köhler & Veluwenkamp, 2024). In this sense, we acknowledge that there are many ways in which one can tease out an account of conceptual functions. However, the present discussion does not hinge on the adoption of any particular account. Our aim here is not to settle the question of what conceptual functions *are* or what they *should be*, but to clarify how the notion of resilience might be understood *given* a broadly functional orientation to conceptual adequacy. The argument proceeds at a general level: whatever one takes conceptual functions to be, it remains meaningful to ask under what conditions the fulfilment of those functions can be sustained under pressure. We thus remain neutral with respect to which (if any) of the existing accounts advanced in the literature captures conceptual functions best, since the general framework of resilience developed in this paper can be instantiated across them, and rather work with a deliberately inclusive understanding of conceptual functions. For the purpose of illustration, in this paper we follow Riggs (2021) in adopting a deflationary functional account, whereby which function(s) a given concept should fulfil is (at least in part) context-dependent and should be determined on a case-by-case basis. We also follow Nado (2021) and Marchiori (2025) in rejecting an all-or-nothing stance towards functional fulfilment. That is to say, concepts are not functionally adequate or inadequate in absolute terms; rather, they are more or less adequate. We will expand on this point in section § 3 when discussing the degrees of conceptual resilience. We recognise that the precise contours of what conceptual adequacy (and, *mutatis mutandis*, resilience) amounts to may vary depending on how one construes these functions, with different functional accounts plausibly leading to resilience being more or less easily achieved or achievable. While this is not the focus of the current paper, future work will explore how specific functional accounts may yield correspondingly distinct models of conceptual resilience.

4 Indeed, Lundgren (2024) argues that, all other things being equal, it is desirable to avoid disruption. In Lundgren’s words, “if we can avoid any conceptual disruption, then that has clear benefits” as “a stable concept is *ceteris paribus* better than a disruptable concept, given that it avoids the need for future conceptual work on that concept” (Lundgren, 2024, p. 2). Two considerations are in order. First, the literature on conceptual disruption does not attribute a positive or negative valence to the phenomenon of disruption in a vacuum, rather suggesting that disruption can be positive, negative, or neutral, depending on how in manifests in a given context (Hopster & Löhr, 2023; Hopster et al., 2023; Löhr, 2023; Marchiori & Scharp, 2024). Indeed, much of the literature is informed by discussions surrounding the neighbouring notion of *social disruption* (Hopster, 2021) in the philosophy of technology, which is itself explicitly noncommittal with regards to the determination of its desirability, which should be assessed on a case-by-case basis (Marchiori et al., 2025; van de Poel et al., 2023). Second, even granting that conceptual disruption may be undesirable (whether in specific contexts or in general), it does not follow that (re-)engineering the disrupted or disruptable concept for resilience will itself be desirable. In this sense, the current paper aims to ground future work on the desirability of (re-)engineering concepts for resilience.

5 There are clear affinities between the present discussion of *conceptual resilience* and Hopster and Löhr’s (2023) account of *conceptual adaptation*. Conceptual adaptation designates an *approach* to conceptual engineering taken in response to (i.e., backward-looking conceptual adaptation), or anticipation of (i.e., forward-looking conceptual adaptation), conceptual disruption, with the aim of “preserving conceptual functions to adapt to new circumstances, irrespective of whether such adaptations qualify as ameliorative” (p. 3). By contrast, conceptual resilience captures a *property* of concepts, i.e., their capacity to remain functionally adequate under pressure. The two notions intersect insofar as conceptual adaptation can be understood as the *means by which* conceptual engineers may (re-)engineer concepts for resilience. Indeed, if one takes resilience to be a desirable property of concepts, conceptual adaptation would constitute the mechanism through which such resilience may be intentionally achieved. Accordingly, this contribution can be framed as a continuation and specification of Hopster & Löhr’s (2023) work on conceptual adaptation, of which we here isolate and develop the proactive, forward-looking dimension. At the same time, conceptual resilience need not follow from conceptual adaptation, as it is a property that concepts can possess independently from conceptual engineering interventions. Ultimately, Lundgren’s (2024) account of *conceptual stability* captures one route to resilience (immutability under pressure) whereas Hopster and Löhr’s notion of *conceptual adaptation* encompasses a wider range of responses, both proactive and reactive, aimed at maintaining or restoring conceptual adequacy relative to a (present or expected) conceptual disruption. The current proposal occupies an intermediate position between these two contributions. We wish to thank one anonymous reviewer for prompting us to expand on this point.

6 Lundgren (2024) clarifies that a distinction could be drawn between conceptual *stability*, which describes concepts which remain adequate *despite* disruptions, and conceptual *undisruptability*, which describes concepts which *cannot* be disrupted. Ultimately, both terms are used as synonyms in Lundgren (2024).

7 The literature on conceptual disruption that this paper builds on is itself either explicitly operating from a functional account of concepts (Marchiori & Scharp, 2024) or nevertheless compatible with such a position (Hopster & Löhr, 2023, Hopster et al., 2023; Löhr, 2022, 2023). While we deem adopting a functional lens to ground conceptual resilience overall desirable, this also leads to possible downstream complications. That is, if concepts are a functional kind (as we assume is the case in this paper), and are therefore identified by their functions, discrepancies in the determination of a given concept’s function(s) by different actors may lead to such actors referring to different concepts to the extent that such function(s) may be understood as not equivalent. That is to say, if one understands the function of good as “providing normative guidance with respect to desirable actions and outcomes”, they may not be willing to accept a concept of good whose function is identified as “providing the standard for ethical behaviour”. While we do not think this is an overriding issue of functionalism, it may introduce challenges, particularly in the form of metalinguistic disputes (e.g., Chalmers, 2011; Plunkett, 2015; Thomasson, 2017; Plunkett & Sundell, 2023). Consequently, conceptual engineers should be mindful of this point.

8 Consider an analogy with virtues. A person’s bravery is not fully apparent unless they are placed in a situation requiring bravery. Similarly, a concept might appear to resist disruption because it has never been challenged in the first place; however, this does not entail that it possesses the capacity to withstand significant challenges.

9 Consider three buildings: the first two buildings are constructed in an earthquake-prone seismic zone, of which only one is constructed with anti-seismic design, and another building is constructed in a non-seismic zone with no special structural reinforcements. Following an earthquake, the building in the earthquake-prone seismic zone which was built without structural reinforcements collapses. We can say that the building was disrupted. However, if both the other buildings remain standing, can we say that they are both resilient? Probably not. The building in the seismic zone demonstrates resilience by withstanding disturbances, while the other building’s stability offers no insight into its ability to endure disturbances.

10 Many thanks to Stefan Buijsman for double-checking this example.

11 It should be noted that Lundgren (2024) does not suggest that conceptual stability should be understood in absolute terms. However, whether or not Lundgren (2024) would endorse a binary account of conceptual conceptual stability is not crucially relevant here. Rather, we are interested in understanding whether this may be a desirable framing, were this to be generalised beyond conceptual stability to the understand of conceptual resilience. Consequently, the criticism of a binary approach to conceptual stability and, relatedly, conceptual resilience might not apply to Lundgren (2024).

12 As already noted, further work is needed to develop a systematic criterion for functional adequacy. However, because our account of conceptual resilience is compatible with different theories of conceptual functions, we refrain from proposing such a standard in this paper, as the relevant criterion for adequacy will likely depend on the functional framework one adopts.

13 It is, of course, possible that some concepts may be functionally adequate and not require (re-)engineering.

14 Our point is that conceptual adequacy is rarely achieved in absolute terms. Even radical proposals (such as Haslanger, 2000, 2020) are themselves subject to subsequent refinement and debate, illustrating that conceptual engineering tends to operate as an ongoing process of continuous (re-)evaluation and improvement, rather than a one-time correction (e.g., Dembroff, 2024; Díaz-León, 2025; Haslanger, 2018; Jenkins, 2016; Marques, 2025; Saul, 2006).

15 Resilience has been widely studied in numerous domains (Grove, 2018), including psychology (Bonanno, 2004; Caldeira & Timmins, 2016; Çuhadar et al., 2016; de Terte et al., 2014), ecology (Cretney, 2014; Gunderson, 2000; Holling, 1973, 1996; Walker, 2023; Walker & Salt, 2012; Walker et al., 2004), disaster studies (Manyena, 2006; Patel et al., 2017), engineering (Bruneau et al., 2003; Hosseini et al., 2016), complex systems (Allen & Holling, 2008), management (Hamel & Välikangas, 2003; Lengnick-Hall et al., 2011; Vogus & Sutcliffe, 2007), urban studies and planning (Leichenko, 2011; Meerow et al., 2016).

16 It should be noted that this is not intended as an exhaustive overview of the conceptualisations of the notion of resilience in the literature on resilience and is merely meant to illustrate the richness of the discussion surrounding the concept of resilience.

17 As already mentioned, despite using similar terminology, our account of conceptual resilience as adaptability differs from Hopster & Löhr’s (2023) notion of conceptual adaptation. Conceptual resilience through adaptability captures the property of concepts to remain adequate despite pressures, whereas conceptual adaptation in Hopster & Löhr’s (2023) sense points to a stance towards the conceptual engineering process. Therefore, a concept may be resilient (in the sense captured by CRA) even in the absence of a conceptual engineering intervention, whereas conceptual adaptation occurs within a conceptual engineering process.

18 We do not intend to suggest that the idea of CRA be found only in the Confucian tradition or only in the broader tradition of Chinese philosophy. Indeed, the CRA frame echoes Moore’s (1903/1959) open question argument, which suggests that any proposed definition of good remains open to normative challenge. While Moore’s concern is meta-ethical, it similarly points to the conceptual openness and indefinability of good.

19 A parallel can be drawn between the Platonic and the Confucian traditions. In *Euthyphro*, Socrates asks the titular character what piety is. Euthyphro answers, “prosecuting those who commit an injustice, such as murder or temple robbery” (*Euthyphro*, 5d/Plato, ed. Reeve, 2012, p. 7). He also says that Zeus chaining his wicked father, Cronos, is piety. Socrates then tells him, “What I urged you to do wasn’t to teach me about one or two of the many pieties, but rather about the form itself, by virtue of which all the pieties are pious. (*Euth.*, 6d-e/Plato, ed. Reeve, 2012, p. 8)” What Socrates implies is that, in order for the multiple and variegated instantiations of piety to be understood as pious, there must be a singular idea of piety that serves as the unchanging standard by which all these instantiations are judged the way they are (i.e., as tokens of piety). It is the explication of this idea in terms of a definition that Socrates asks from Euthyphro. Now, consider the following examples in the *Analects*: “Zhonggong asked about Goodness [i.e., Ren 仁]. The Master [Confucius] said, ‘When in public, comport yourself as if you were receiving an important guest, and in your management of the common people, behave as if you were overseeing a great sacrifice.’” (*Analects* 12.2/Confucius, trans. Slingerland, 2003, p. 126); “Sima Niu asked about Goodness. The Master said, ‘The Good person is hesitant to speak.’” (*Analects* 12.3/Confucius, trans. Slingerland, 2003, p. 126); and “Fan Chi asked about Goodness. The Master said, ‘Care for others.’” (*Analects* 12.22/Confucius, trans. Slingerland, 2003, p. 136).

20 Confucius’ refusal to provide universal or exhaustive definitions of ethical ideas such as Ren is also understood by scholars as part of his way of providing optimal practical moral guidance to his students (Xiao, 2008, Rogacz, 2022). That is to say, Confucius considered the activity of exhaustively defining concepts to be an unhelpful exercise since it cannot be ultimately achieved and can potentially distract students from the more important task of cultivating ethical virtue.02

21 Three brief concluding considerations are in order. First, we do not aim to be prescriptive and suggest that any one frame (CRI or CRA) should be preferred over the other. Second, and relatedly, while concepts resulting from CRI and CRA approaches to conceptual (re-)engineering may exhibit resilience in similar ways, they may differ in their philosophical orientation. For example, the position advanced in Lundgren (2024) explicitly distances itself from bottom-up approaches, more akin to a CRA frame, arguing that they risk excessive relativity (Lundgren, 2024, p. 10). Third and last, it should be noted that different theoretical commitments may lead to different interpretations of what displays resilience, whether concepts themselves or conceptions. As already discussed, the account of conceptual resilience put forth in this paper can be applied across different theoretical accounts of concepts. Depending on one’s preferred view of concepts, resilience may be understood as operating at the level of concepts or at the level of conceptions. For example, is it the concept good that exhibits resilience, or rather specific conceptions of good, such as Platonic-good or Confucian-good? On some accounts, it may be preferable to say that the concept of good itself is resilient, while other accounts may find it more accurate to describe resilience as a property of conceptions (e.g., Platonic-good or Confucian-good). Accordingly, conceptual resilience is a property that can be instantiated across different theories of concepts and at different levels (concepts or conceptions).

## References

[CR1] Alexander, D. E. (2013). Resilience And disaster risk reduction: An etymological journey. *Natural Hazards and Earth System Sciences*, *13*(11), 2707–2716.

[CR2] Allen, C. R., & Holling, C. S. (Eds.). (2008). *Discontinuities in ecosystems and other complex systems*. Columbia University.

[CR3] Ananny, M., & Crawford, K. (2018). Seeing without knowing: Limitations of the transparency ideal and its application to algorithmic accountability. *New Media & Society*, *20*(3), 973–989.

[CR4] Anderson, R. T. (2013). Marriage: What it is, why it matters, and the consequences of redefining it. *Heritage Foundation Backgrounder*, *2775*, 1–12.

[CR5] Ashmore, R. (2004). Word and gesture: On Xuan-School hermeneutics of the analects. *Philosophy East and West*, *54*(4), 458–488. https://www.jstor.org/stable/4148015

[CR6] Bajohr, H. (2024). Writing at a distance: Notes on authorship and artificial intelligence. *German Studies Review*, *47*(2), 315–337.

[CR7] Berkes, F., & Folke, C. (Eds.). (1998). *Linking social and ecological systems: management practices and social mechanisms for building resilience*. Cambridge University Press.

[CR8] Bimo, S. (2024). A brief history of authorship. *The Routledge Handbook of AI and Literature*, 49.

[CR9] Bonanno, G. A. (2004). Loss, trauma, and human resilience: Have we underestimated the human capacity to thrive after extremely aversive events? *American Psychologist*, *59*(1), 20–28. 10.1037/0003-066X.59.1.20 14736317 10.1037/0003-066X.59.1.20

[CR10] Briguglio, L., Cordina, G., Farrugia, N., & Vella, S. (2009). Economic vulnerability and resilience: Concepts and measurements. *Oxford Development Studies*, *37*(3), 229–247.

[CR11] Bruneau, M., Chang, S. E., Eguchi, R. T., Lee, G. C., O’Rourke, T. D., Reinhorn, A. M., & Von Winterfeldt, D. (2003). A framework to quantitatively assess and enhance the seismic resilience of communities. *Earthquake Spectra*, *19*(4), 733–752.

[CR12] Caldeira, S., & Timmins, F. (2016). Resilience: Synthesis of concept analyses and contribution to nursing classifications. *International Nursing Review*, *63*(2), 191–199. 27029400 10.1111/inr.12268

[CR13] Cappelen, H. (2018). *Fixing language: An essay on conceptual engineering*. Oxford University Press.

[CR14] Cappelen, H., & Plunkett, D. (2020). A guided tour of conceptual engineering and conceptual ethics. In A. Burgess, H. Cappelen, & D. Plunkett (Eds.), *Conceptual engineering and conceptual ethics*. Oxford University Press.

[CR116] Carpenter, S., Walker, B., Anderies, J. M., & Abel, N. (2001). From metaphor to measurement: resilience of what to what? *Ecosystems*, *4*(8), 765–781.

[CR16] Chalmers, D. J. (2011). Verbal disputes. *Philosophical Review,**120*(4), 515–566.

[CR17] Chalmers, D. J. (2020). What is conceptual engineering and what should it be? *Inquiry*. 10.1080/0020174X.2020.1817141

[CR18] Coeckelbergh, M., & Gunkel, D. J. (2025). *Communicative AI: A critical introduction to large Language models*. John Wiley & Sons.

[CR19] Comporti, M. (2009). *Fatti illeciti: Le responsabilità oggettive.* Giuffrè Editore.

[CR20] Confucius. (2003). *Analects: With sections from traditional commentaries*. (Slingerland, E., Trans.) Hackett.

[CR21] Conrey, J. B. (2003). The Riemann hypothesis. *Notices of the AMS,**50*(3), 341–353.

[CR22] Cretney, R. (2014). Resilience for whom? Emerging critical geographies of socio-ecological resilience. *Geography Compass*, *8*(9), 627–640.

[CR129] Cuhadar, D., Tanriverdi, D., Pehlivan, M., Kurnaz, G., & Alkan, S. (2016). Determination of the psychiatric symptoms and psychological resilience levels of hematopoietic stem cell transplant patients and their relatives. *European Journal of Cancer Care*, *25*(1), 112–121. 25040559 10.1111/ecc.12219

[CR25] da Fonseca, A. T., de Vaz Sequeira, E., & Barreto Xavier, L. (2023). Liability for AI Driven Systems. *Multidisciplinary Perspectives on Artificial Intelligence and the Law* (pp. 299–317). Springer International Publishing.

[CR23] D’Ambrosio, P. (2016). Guo Xiang on Self-so knowledge. *Asian Philosophy*, *26*(2), 119–132. 10.1080/09552367.2016.1163774

[CR24] D’Ambrosio, P. (2019). Wang bi’s commentary on the *Analects*: A Confucian-Daoist critique of effable morality. *Philosophy East and West*, *69*(2), 357–375. 10.1353/pew.2019.0030

[CR29] Díaz-León, E. (2025). *The Metaphysics of Gender*. Cambridge University Press.

[CR28] Dembroff, R. (2024). Intersection is not identity, or how to identify overlapping systems of injustice. In R. Chang & A. Srinivasan (Eds.), *Conversations in Philosophy, Law, and Politics* (pp. 383–400). Oxford University Press.

[CR27] de Terte, I., Stephens, C., & Huddleston, L. (2014). The development of a three-part model of psychological resilience. *Stress and Health,**30*(5), 416–424. 10.1002/smi.2625 25476966 10.1002/smi.2625

[CR30] Dworkin, G. (1988). *The Theory and Practice of Autonomy*. Cambridge University Press.

[CR15] Čerka, P., Grigienė, J., & Sirbikytė, G. (2015). Liability for damages caused by artificial intelligence. *Computer Law & Security Review*, *31*(3), 376–389.

[CR31] Fineman, M. A. (2001). Why marriage? *Virginia Journal of Social Policy & the Law,**9*, 239.

[CR32] Fineman, M. A. (2006). The meaning of marriage. *Marriage proposals: Questioning a legal status,**29*, 43–57.

[CR33] Floridi, L. (2023). *The Ethics of Artificial Intelligence: Principles, Challenges, and Opportunities*. Oxford University Press.

[CR34] Floridi, L. (2025). Distant writing: Literary production in the age of artificial intelligence. *Minds and Machines*, *35*(3), 1–26. 10.1007/s11023-025-09738-9 PMC12307450 40746976

[CR35] Folke, C., Carpenter, S. R., Walker, B., Scheffer, M., Chapin, T., & Rockström, J. (2010). Resilience thinking: integrating resilience, adaptability and transformability. *Ecology and Society*, *15*(4).

[CR36] Gallie, W. B. (1955). Essentially contested concepts. *Proceedings of the Aristotelian Society,**56*, 167–198.

[CR37] Gervais, D. J. (2025). Artificial intellectual property. *Chicago-Kent Law Review,**100*, 265.

[CR38] Goetze, T. S. (2022). Mind the gap: autonomous systems, the responsibility gap, and moral entanglement. In *Proceedings of the 2022 ACM Conference on Fairness, Accountability, and Transparency* (pp. 390–400).

[CR126] Grove, K. (2018). Resilience as Essentially Contested Concept. In K. Grove, *Resilience* (1st ed., pp. 30–64). Routledge. 10.4324/9781315661407-2

[CR39] Gunderson, L. H. (2000). Ecological resilience in theory and application. *Annual Review of Ecology and Systematics,**31*(1), 425–439.

[CR40] Hamel, G., & Välikangas, L. (2003). The quest for resilience. *Harvard Business Review*, *81*(9), 52–63. 12964393

[CR41] Hardy, G. H., & Wright, E. M. (1960). *An Introduction to the Theory of Numbers* (4th ed). Oxford University Press.

[CR42] Haslanger, S. (2000). Gender and race: (What) are they? (What) do we want them to be? *Noûs*, *34*(1), 31–55.

[CR43] Haslanger, S. (2018). Social explanation: Structures, Stories, and Ontology. A reply to Díaz León, Saul, and sterken. *Disputatio: International Journal of Philosophy*, *10*(50).

[CR44] Haslanger, S. (2020). Going on, not in the same way. In A. Burgess, H. Cappelen, D. Plunkett (Eds.), *Conceptual engineering and conceptual ethics* (pp. 230–260).

[CR121] Hill, E., Wial, H., & Wolman, H. (2008).Exploring regional economic resilience. UC Berkeley IURD Working Paper Series. https://escholarship.org/uc/item/7fq4n2cv

[CR45] Hindriks, F., & Veluwenkamp, H. (2023). The risks of autonomous machines: From responsibility gaps to control gaps. *Synthese,**201*(1), 21.

[CR46] Holling, C. S. (1973). Resilience and stability of ecological systems. *Annual Review of Ecology and Systematics,**4*(1), 1–23.

[CR47] Holling, C. S. (1996). Engineering resilience versus ecological resilience. In P. C. Schulze (Ed.), *Engineering within Ecological Constraints* (pp. 31–44). National Academy Press.

[CR48] Hopster, J. (2021). What are socially disruptive technologies? *Technology in Society*, *67*, 101750.

[CR49] Hopster, J. (2024a). Is ARTIFICIËLE INTELLIGENTIE Een defectief concept? *Algemeen Nederlands Tijdschrift Voor Wijsbegeerte*, *116*(4), 337–351.

[CR51] Hopster, J., Brey, P., Klenk, M. B. O. T., Löhr, G., Marchiori, S., Lundgren, B., & Scharp, K. (2023). Conceptual disruption and the ethics of technology. In I. van de Poel, L. Frank, J. Hermann, J. Hopster, D. Lenzi, S. Nyholm, B. Taebi, & E. Ziliotti (Eds.), *Ethics of Socially Disruptive Technologies: An Introduction* (pp. 141–162). Open Book Publishers. 10.11647/obp.0366.06

[CR50] Hopster, J. K. G. (2024b). Socially disruptive technologies and epistemic injustice. *Ethics and Information Technology*, *26*(1), 14.

[CR52] Hosseini, S., Barker, K., & Ramirez-Marquez, J. E. (2016). A review of definitions and measures of system resilience. *Reliability Engineering & System Safety,**145*, 47–61.

[CR53] Huang, B., Chen, C., & Shu, K. (2025). Authorship attribution in the era of llms: Problems, methodologies, and challenges. *ACM SIGKDD Explorations Newsletter*, *26*(2), 21–43. 10.1145/3715073.3715076 PMC12019761 40276161

[CR54] Isaac, M. G., Koch, S., & Nefdt, R. (2022). Conceptual engineering: A road map to practice. *Philosophy Compass,**17*(10), Article e12879.

[CR55] Ivy, B. P. U., Mandiwa, P., & Kumar, M. (2012). A modified RSA cryptosystem based on ’n’ prime numbers. *International Journal Of Engineering And Computer Science,**1*(2), 63–66.

[CR56] Jacobs, M. (1999). Sustainable development as a contested concept. In A. Dobson (Ed.), *Fairness and Futurity* (pp. 21–45). Oxford University Press.

[CR57] Jameson, G. J. O. (2003). *The prime number theorem*. Cambridge University Press.

[CR58] Jenkins, K. (2016). Amelioration and inclusion: Gender identity and the concept of woman. *Ethics*, *126*(2), 394–421.

[CR118] Keck, M., & Sakdapolrak, P. (2013). What is social resilience? Lessons learned and ways forward. *Erdkunde*, *67*(1), 5–19.

[CR59] Köhler, S., & Veluwenkamp, H. (2024). Conceptual engineering: For what matters. *Mind*, *133*(530), 400–427. 10.1093/mind/fzad064

[CR60] Lalumera, E. (2009). *Cosa Sono i Concetti*. Editore Laterza.

[CR61] Leichenko, R. (2011). Climate change and urban resilience. *Current Opinion in Environmental Sustainability*, *3*(3), 164–168.

[CR62] Lengnick-Hall, C. A., Beck, T. E., & Lengnick-Hall, M. L. (2011). Developing a capacity for organizational resilience through strategic human resource management. *Human Resource Management Review,**21*(3), 243–255.

[CR63] Löhr, G. (2022). Linguistic interventions and the ethics of conceptual disruption. *Ethical Theory and Moral Practice,**25*, 835–849. 10.1007/s10677-022-10321-9

[CR64] Löhr, G. (2023). Conceptual disruption and 21st century technologies: A framework. *Technology in Society,**74*, Article 102327. 10.1016/j.techsoc.2023.102327

[CR65] Lozano-Robledo, Á. (2019). *Number theory and geometry: an introduction to arithmetic geometry* (Vol. 35). American Mathematical Society.

[CR66] Lundgren, B. (2024). Undisruptable or stable concepts: Can we design concepts that can avoid conceptual disruption, normative critique, and counterexamples? *Ethics and Information Technology,**26*(2), Article 33. 10.1007/s10676-024-09767-5

[CR67] Luthar, S. S., Cicchetti, D., & Becker, B. (2000). The construct of resilience: A critical evaluation and guidelines for future work. *Child Development,**71*(3), 543–562. 10953923 10.1111/1467-8624.00164 PMC1885202

[CR68] Mackenzie, C., & Stoljar, N. (Eds.). (2000). *Relational autonomy: feminist perspectives on autonomy, agency, and the social self*. Oxford University Press.

[CR122] Magis, K. (2010). Community resilience: An indicator of social sustainability. *Society and Natural Resources*, *23*(5), 401–416.

[CR69] Manyena, S. B. (2006). The concept of resilience revisited. *Disasters,**30*(4), 434–450. 10.1111/j.0361-3666.2006.00331.x 17100752

[CR123] Manyena, S. B., O'Brien, G., O'Keefe, P., & Rose, J. (2011). Disaster resilience: a bounce back or bounce forward ability? Local Environment,16(5).

[CR70] Marchiori, S. (2025). Conceptual affordances. (How) should they inform conceptual engineering? *Synthese*.

[CR125] Marchiori, S., Hopster, J. K., Puzio, A., Riemsdijk, M. B. V., Kraaijeveld, S. R., Lundgren, B., Viehoff, J., & Frank, L. E. (2025). A social disruptiveness-based approach to AI governance: complementing the risk-based approach of the AI Act. *Science and engineering ethics*, 31(5), 25. 10.1007/s11948-025-00545-0 PMC12391233 40864303

[CR71] Marchiori, S., & Scharp, K. (2024). What is conceptual disruption? *Ethics and Information Technology,**26*(1), Article 18.

[CR72] Margolis, E., & Laurence, S. (2023). Concepts. In E. N. Zalta & U. Nodelman (Eds.), *The Stanford Encyclopedia of Philosophy (Fall 2023 Edition)*. Available at https://plato.stanford.edu/archives/fall2023/entries/concepts

[CR73] Marques, T. (2025). Representing or shaping reality? What class can teach about woman. *New perspectives on conceptual Engineering-Volume 2: Across philosophy* (pp. 95–113). Springer Nature Switzerland.

[CR74] Matthias, A. (2004). The responsibility gap: Ascribing responsibility for the actions of learning automata. *Ethics and Information Technology*, *6*(3), 175–183.

[CR75] Maynard, J. (2019). The twin prime conjecture. *Japanese Journal of Mathematics*, *14*(2), 175–206.

[CR76] McDonald, L. (2023). AI systems and liability: An assessment of the applicability of strict liability & a case for limited legal personhood for AI. *St Andrews Law Journal,**3*(1), 5–21.

[CR77] Meerow, S., Newell, J. P., & Stults, M. (2016). Defining urban resilience: A review. *Landscape and Urban Planning*, *147*, 38–49.

[CR78] Millikan, R. G. (1989). In defense of proper functions. *Philosophy of Science,**56*(2), 288–302.

[CR128] Moore, G. E. (1903/1959). *Principia ethica*. Cambridge University Press.

[CR79] Nado, J. (2021). Conceptual engineering, truth, and efficacy. *Synthese*, *198*, 1507–1527. 10.1007/s11229-019-02096-x

[CR80] Nathanson, M. B. (1996). *Additive Number Theory* (Vol. 164). Springer.

[CR81] Nickel, P. J. (2020). Disruptive innovation and moral uncertainty. *Nanoethics,**14*(3), 259–269.

[CR82] Nissenbaum, H. (2009). *Privacy in Context: Technology, Policy, and the Integrity of Social Life*. Stanford University Press.

[CR83] Nissenbaum, H. (2011). A contextual approach to privacy online. *Daedalus*, *140*(4), 32–48.

[CR84] Nissenbaum, H. (2018). Respecting context to protect privacy: Why meaning matters. *Science and Engineering Ethics*, *24*(3), 831–852. 26164733 10.1007/s11948-015-9674-9

[CR117] Norris, F. H., Stevens, S. P., Pfefferbaum, B., Wyche, K. F., & Pfefferbaum, R. L. (2008). Community resilience as a metaphor, theory, set of capacities, and strategy for disaster readiness. *American Journal of Community Psychology*, *41*(1), 127–150. 18157631 10.1007/s10464-007-9156-6

[CR85] Patel, S. S., Rogers, M. B., Amlôt, R., & Rubin, G. J. (2017). What do we mean by “community resilience”? A systematic literature review of how it is defined in the literature. *PLoS Currents,**9*, Article ecurrents.dis.db775aff25efc5ac4f0660ad9c9f7db2. 29188132 10.1371/currents.dis.db775aff25efc5ac4f0660ad9c9f7db2 PMC5693357

[CR86] Pimm, S. L. (1984). The complexity and stability of ecosystems. *Nature,**307*(5949), 321–326.

[CR87] Plato. (2012). Euthyphro. In C. D. Reeve (Ed.), *A Plato reader* (pp. 1–20). Hackett.

[CR88] Plunkett, D. (2015). Which concepts should we use? Metalinguistic negotiations and the methodology of philosophy. *Inquiry : A Journal of Medical Care Organization, Provision and Financing*, *58*(7–8), 828–874.

[CR89] Plunkett, D., & Sundell, T. (2023). Varieties of metalinguistic negotiation. *Topoi*, *42*(4), 983–999.

[CR90] Prinzing, M. (2018). The revisionist’s rubric: Conceptual engineering and the discontinuity objection. *Inquiry : A Journal of Medical Care Organization, Provision and Financing*, *61*(8), 854–880.

[CR91] Queloz, M. (2019). The points of concepts: Their types, tensions, and connections. *Canadian Journal of Philosophy*, *49*(8), 1122–1145.

[CR92] Queloz, M. (2022). Function-based conceptual engineering and the authority problem. *Mind*, *131*(524), 1247–1278.

[CR93] Rawls, J. (1971). *A Theory of Justice.* Harvard University Press. 10.2307/j.ctvjf9z6v

[CR94] Riggs, J. (2021). Deflating the functional turn in conceptual engineering. *Synthese*, *199*(3–4), 11555–11586. 10.1007/s11229-021-03302-5

[CR127] Rogacz, D. (2022). Operating with Names: Operational Definitions in the Analects and Beyond. *Dao*, *21*, 19–35. 10.1007/s11712-021-09813-9

[CR119] Rose, A. (2004). Defining and measuring economic resilience to disasters. Disaster Prevention and Management. *An International Journal*, *13*(4), 307–314.

[CR120] Rose, A. (2007). Economic resilience to natural and man-made disasters: Multidisciplinary origins and contextual dimensions. *Environmental Hazards*, *7*(4), 383–398.

[CR95] Rutter, M. (1987). Psychosocial resilience and protective mechanisms. *American Journal of Orthopsychiatry*, *57*(3), 316–331. 3303954 10.1111/j.1939-0025.1987.tb03541.x

[CR96] Saidak, F. (2006). On goldbach’s conjecture for integer polynomials. *The American Mathematical Monthly*, *113*(6), 541–545.

[CR26] Santoni de Sio, F., & Mecacci, G. (2021). Four responsibility gaps with artificial intelligence: Why they matter and how to address them. *Philosophy & Technology,**34*(4), 1057–1084.

[CR97] Saul, J. (2006). II—Jennifer saul: Gender and race. *Aristotelian society supplementary volume* (Vol. 80, pp. 119–143). Oxford University Press. 1.

[CR98] Selberg, A. (1949). An elementary proof of dirichlet’s theorem about primes in an arithmetic progression. *Annals of Mathematics*, *50*(2), 297–304.

[CR99] Shemanske, T. R. (2017). *Modern Cryptography and Elliptic Curves* (Vol. 83). American Mathematical Society.

[CR100] Simion, M., & Kelp, C. (2020). Conceptual innovation, function first. *Noûs*, *54*(4), 985–1002.

[CR101] Solove, D. J. (2010). *Understanding Privacy*. Harvard University Press.

[CR102] Tao, T. (2011). Structure and randomness in the prime numbers. *An Invitation to Mathematics: From Competitions to Research* (pp. 1–7). Springer Berlin Heidelberg.

[CR103] Thomasson, A. (2020). A pragmatic method for normative conceptual work. In A. Burgess, H. Cappelen, D. Plunkett (Eds.), *Conceptual engineering and conceptual ethics* (pp. 435–458).

[CR104] Thomasson, A. (2021). Conceptual engineering: When do we need it? How can we do it? *Inquiry : A Journal of Medical Care Organization, Provision and Financing*. 10.1080/0020174X.2021.2000118

[CR105] Thomasson, A. (2022). How should we think about linguistic function? *Inquiry : A Journal of Medical Care Organization, Provision and Financing*. 10.1080/0020174X.2022.2074886

[CR106] Thomasson, A. L. (2017). Metaphysical disputes and metalinguistic negotiation. *Analytic Philosophy,**58*(1), 1–28.

[CR107] van de Poel, I., Frank, L., Hermann, J., Hopster, J., Lenzi, D., Nyholm, S., Taebi, B., & Ziliotti, E. (Eds.). (2023). *Ethics of Socially Disruptive Technologies: An Introduction.* Open Book Publishers. 10.11647/OBP.0366

[CR108] Vogus, T. J., & Sutcliffe, K. M. (2007). Organizational resilience: towards a theory and research agenda. In *2007 IEEE international conference on systems, man and cybernetics* (pp. 3418–3422). IEEE.

[CR110] Walker, B., Holling, C. S., Carpenter, S. R., & Kinzig, A. (2004). Resilience, adaptability and transformability in social–ecological systems. *Ecology and society,**9*(2), Article 5.

[CR109] Walker, B., & Salt, D. (2012). *Resilience thinking: Sustaining ecosystems and people in a changing world.* Island Press.

[CR111] Walker, S. (2023). *Design for Resilience*. MIT Press.

[CR112] Wendehorst, C. (2020). Strict liability for AI and other emerging technologies. *Journal of European Tort Law*, *11*(2), 150–180.

[CR124] Westley, F., Olsson, P., Folke, C., Homer-Dixon, T., Vredenburg, H., Loorbach, D., Thompson, J., Nilsson, M., Lambin, E., Sendzimir, J., Banerjee, B., Galaz, V., & Van Der Leeuw, S. (2011). Tipping toward sustainability: Emerging pathways of transformation. *Ambio*, *40*(7), 762–780. 22338714 10.1007/s13280-011-0186-9 PMC3357751

[CR113] Xiao, X. (2008). Ancient Greek and Chinese patterns of definition: A comparative study. *Intercultural Communication Studies*, *7*(2), 61–77.

[CR114] Young, I. M. (1990). *Justice and the Politics of Difference.* Princeton University Press.

[CR115] Zhou, X., & Tang, X. (2011). Research and implementation of RSA algorithm for encryption and decryption. In *Proceedings of 2011 6th international forum on strategic technology* (Vol. 2, pp. 1118–1121). IEEE.

